# Supplementary material for: An open-source framework for large-scale, flexible evaluation of biomedical text mining systems
Source: J Biomed Discov Collab. 2008 Jan 29;3:1. doi: 10.1186/1747-5333-3-1 (PMC2276192; doi:10.1186/1747-5333-3-1)
Supplement: Additional file 1 — Evaluation platform source code. The additional file contains the software evaluation framework discussed in this paper. The most current release can be downloaded from SourceForge.net [1]. See the accompanying README file for instructions on installation and use. Also included in the distribution is a collection of UIMA wrappers for some commonly used BioNLP tools and annotated corpora. [file 1747-5333-3-1-S1.zip › bionlp_uima_repository-1.0.1/docs/api/edu/uchsc/ccp/uima/annotation/CCPDocumentSection.html]

CCPDocumentSection (Center for Computational Pharmacology Util/UIMA API)


|  |  |  |
| --- | --- | --- |
| **Overview** \| **Package** \| **Class** \| **Tree** \| **Deprecated** \| **Index** \| **Help** | |  |
| **PREV CLASS**   **NEXT CLASS** | **FRAMES**    **NO FRAMES**     **All Classes** |  |
| SUMMARY: NESTED \| FIELD \| CONSTR \| METHOD | DETAIL: FIELD \| CONSTR \| METHOD |  |


---


## edu.uchsc.ccp.uima.annotation Class CCPDocumentSection

```
java.lang.Object
  org.apache.uima.cas.impl.FeatureStructureImpl
      org.apache.uima.jcas.cas.TOP
          org.apache.uima.jcas.cas.AnnotationBase
              org.apache.uima.jcas.tcas.Annotation
                  edu.uchsc.ccp.uima.annotation.CCPAnnotation
                      edu.uchsc.ccp.uima.annotation.CCPDocumentAnnotation
                          edu.uchsc.ccp.uima.annotation.CCPDocumentSection
```

**All Implemented Interfaces:**: Cloneable, org.apache.uima.cas.AnnotationBaseFS, org.apache.uima.cas.FeatureStructure, org.apache.uima.cas.text.AnnotationFS

---

``` public class CCPDocumentSection extends CCPDocumentAnnotation ```

The Document Section annotation allows for an entire document section to be annotated as such, and stores section-specific information such as the section identifier.
Updated by JCasGen Tue Aug 28 16:44:10 MDT 2007
XML source: /home/williamb/eclipse/RELEASED\_SOURCE\_CODE/descriptors/typeSystem/CCPTypeSystem.xml

---

| **Field Summary** | |
| --- | --- |
| `static int` | `type` |
| `static int` | `typeIndexID` |

**Fields inherited from class org.apache.uima.jcas.cas.TOP**

`addr, jcasType`


| **Constructor Summary** | |
| --- | --- |
| `protected` | `CCPDocumentSection()`             Never called. |
|  | `CCPDocumentSection(int addr, org.apache.uima.jcas.cas.TOP_Type type)`             Internal - constructor used by generator |
|  | `CCPDocumentSection(org.apache.uima.jcas.JCas jcas)` |
|  | `CCPDocumentSection(org.apache.uima.jcas.JCas jcas, int begin, int end)` |


| **Method Summary** | |
| --- | --- |
| `int` | `getSectionID()`             getter for sectionID - gets The section ID for this document section, e.g. |
| `int` | `getSectionLength()`             getter for sectionLength - gets The length of this document section (in characters). |
| `String` | `getSectionName()`             getter for sectionName - gets The name of this document section, e.g. |
| `org.apache.uima.jcas.cas.FSArray` | `getSubSections()`             getter for subSections - gets Allows for direct linking to CCPDocumentSubSections residing in this document section. |
| `CCPDocumentSubSection` | `getSubSections(int i)`             indexed getter for subSections - gets an indexed value - Allows for direct linking to CCPDocumentSubSections residing in this document section. |
| `int` | `getTypeIndexID()` |
| `void` | `setSectionID(int v)`             setter for sectionID - sets The section ID for this document section, e.g. |
| `void` | `setSectionLength(int v)`             setter for sectionLength - sets The length of this document section (in characters). |
| `void` | `setSectionName(String v)`             setter for sectionName - sets The name of this document section, e.g. |
| `void` | `setSubSections(org.apache.uima.jcas.cas.FSArray v)`             setter for subSections - sets Allows for direct linking to CCPDocumentSubSections residing in this document section. |
| `void` | `setSubSections(int i, CCPDocumentSubSection v)`             indexed setter for subSections - sets an indexed value - Allows for direct linking to CCPDocumentSubSections residing in this document section. |

**Methods inherited from class org.apache.uima.jcas.tcas.Annotation**

`getBegin, getCoveredText, getEnd, getStart, setBegin, setEnd`

**Methods inherited from class org.apache.uima.jcas.cas.AnnotationBase**

`getSofa, getView`

**Methods inherited from class org.apache.uima.jcas.cas.TOP**

`addToIndexes, addToIndexes, equals, getAddress, getCAS, getCASImpl, getLowLevelCas, hashCode, removeFromIndexes, removeFromIndexes`

**Methods inherited from class org.apache.uima.cas.impl.FeatureStructureImpl**

`clone, getBooleanValue, getByteValue, getDoubleValue, getFeatureValue, getFeatureValueAsString, getFloatValue, getIntValue, getLongValue, getShortValue, getStringValue, getType, prettyPrint, prettyPrint, prettyPrint, setBooleanValue, setByteValue, setDoubleValue, setFeatureValue, setFeatureValueFromString, setFloatValue, setIntValue, setLongValue, setShortValue, setStringValue, toString, toString`

**Methods inherited from class java.lang.Object**

`finalize, getClass, notify, notifyAll, wait, wait, wait`

**Methods inherited from interface org.apache.uima.cas.AnnotationBaseFS**

`getView`

**Methods inherited from interface org.apache.uima.cas.FeatureStructure**

`clone, equals, getBooleanValue, getByteValue, getCAS, getDoubleValue, getFeatureValue, getFeatureValueAsString, getFloatValue, getIntValue, getLongValue, getShortValue, getStringValue, getType, hashCode, setBooleanValue, setByteValue, setDoubleValue, setFeatureValue, setFeatureValueFromString, setFloatValue, setIntValue, setLongValue, setShortValue, setStringValue`

**Field Detail**

### typeIndexID

```
public static final int typeIndexID
```

---


### type

```
public static final int type
```


**Constructor Detail**

### CCPDocumentSection

```
protected CCPDocumentSection()
```

:   Never called. Disable default constructor

---


### CCPDocumentSection

```
public CCPDocumentSection(int addr,
                          org.apache.uima.jcas.cas.TOP_Type type)
```

:   Internal - constructor used by generator

---


### CCPDocumentSection

```
public CCPDocumentSection(org.apache.uima.jcas.JCas jcas)
```

---


### CCPDocumentSection

```
public CCPDocumentSection(org.apache.uima.jcas.JCas jcas,
                          int begin,
                          int end)
```


**Method Detail**

### getTypeIndexID

```
public int getTypeIndexID()
```

:   **Overrides:**: `getTypeIndexID` in class `CCPDocumentAnnotation`

---


### getSectionID

```
public int getSectionID()
```

:   getter for sectionID - gets The section ID for this document section, e.g. 1 = "Title" 2= "Abstract"

---


### setSectionID

```
public void setSectionID(int v)
```

:   setter for sectionID - sets The section ID for this document section, e.g. 1 = "Title" 2= "Abstract"

---


### getSectionLength

```
public int getSectionLength()
```

:   getter for sectionLength - gets The length of this document section (in characters).

---


### setSectionLength

```
public void setSectionLength(int v)
```

:   setter for sectionLength - sets The length of this document section (in characters).

---


### getSectionName

```
public String getSectionName()
```

:   getter for sectionName - gets The name of this document section, e.g. Title, Abstract, Introduction, etc.

---


### setSectionName

```
public void setSectionName(String v)
```

:   setter for sectionName - sets The name of this document section, e.g. Title, Abstract, Introduction, etc.

---


### getSubSections

```
public org.apache.uima.jcas.cas.FSArray getSubSections()
```

:   getter for subSections - gets Allows for direct linking to CCPDocumentSubSections residing in this document section.

---


### setSubSections

```
public void setSubSections(org.apache.uima.jcas.cas.FSArray v)
```

:   setter for subSections - sets Allows for direct linking to CCPDocumentSubSections residing in this document section.

---


### getSubSections

```
public CCPDocumentSubSection getSubSections(int i)
```

:   indexed getter for subSections - gets an indexed value - Allows for direct linking to CCPDocumentSubSections residing in this document section.

---


### setSubSections

```
public void setSubSections(int i,
                           CCPDocumentSubSection v)
```

:   indexed setter for subSections - sets an indexed value - Allows for direct linking to CCPDocumentSubSections residing in this document section.


---


|  |  |  |
| --- | --- | --- |
| **Overview** \| **Package** \| **Class** \| **Tree** \| **Deprecated** \| **Index** \| **Help** | |  |
| **PREV CLASS**   **NEXT CLASS** | **FRAMES**    **NO FRAMES**     **All Classes** |  |
| SUMMARY: NESTED \| FIELD \| CONSTR \| METHOD | DETAIL: FIELD \| CONSTR \| METHOD |  |


---
